# Mental Health Changes in US Transgender Adults Beginning Hormone Therapy Via Telehealth: Longitudinal Cohort Study

**DOI:** 10.2196/64017

**Published:** 2025-02-14

**Authors:** Jae Downing Corman, Jaclyn M W Hughto, Theresa I Shireman, Kellan Baker, Kate Steinle, Michelle Forcier

**Affiliations:** 1 FOLX Health Boston, MA United States; 2 Brown University Providence, RI United States; 3 Whitman-Walker Health Washington, DC United States

**Keywords:** transgender, LGBT persons, telehealth, depression, anxiety, suicide, mental health, adult, virtual care, longitudinal cohort study, gender-affirming hormone therapy, United States, observational study, adolescent, mobile health

## Abstract

**Background:**

Gender-affirming hormone therapy (GAHT) has shown potential for improving mental health outcomes among transgender and gender-diverse adults. How clinical outcomes change among adults receiving GAHT via telehealth across the United States is not well known.

**Objective:**

This study evaluated the relationship between initiating GAHT via a telehealth clinic and changes in depression, anxiety, and suicide ideation over a 3-month period.

**Methods:**

This cohort study evaluated the relationship between initiating GAHT via a telehealth clinic and changes in mental health over a 3-month period. Data were collected at baseline and 3 months later among adults who had their first GAHT visit between August and November 2023. The study included adults aged 18 years and older initiating GAHT for the first time, with a final sample of 342 adults across 43 states (192 initiated estrogen and 150 initiated testosterone therapy). The primary outcomes were depression symptoms using the Patient Health Questionnaire-9 (PHQ-9), anxiety symptoms using the General Anxiety Disorder-7 (GAD-7), and suicide ideation in the past 2 weeks.

**Results:**

Before GAHT initiation, 40% (136/342) of participants reported depression (PHQ-9 ≥10), 36% (120/342) reported anxiety (GAD-7 ≥8), and 25% (91/342) reported suicidal ideation. By follow-up, significant reductions were observed in PHQ-9 (−2.4, 95% CI −3.0 to −1.8) and GAD-7 scores (−1.5, 95% CI −2.0 to −1.0). Among those with elevated symptoms, 40% (48/120) to 42% (56/133) achieved a clinically meaningful response (≥50% reduction in baseline scores), and 27% (36/133) to 28% (33/120) achieved remission (PHQ-9 or GAD-7 score <5). Of those with suicide ideation at baseline, 60% (50/83) had none at follow-up.

**Conclusions:**

This study highlights the important relationship between telehealth-delivered GAHT and mental health, emphasizing the importance of accessible and timely care.

## Introduction

Transgender and gender-diverse (TGD) individuals have poorer mental health outcomes than their cisgender peers, including depression, anxiety, and suicide ideation [1-3]. TGD individuals consistently face significant barriers to accessing gender-affirming medical and surgical treatments, leading many to delay or altogether forgo necessary care [3]. These challenges include difficulty in locating providers who are affirming and knowledgeable, financial barriers to paying for care, and frequent discrimination in health care settings [4-6]. Despite legislation against gender identity discrimination [7], an unprecedented number of states enacted banned medical care for transgender people between 2021 and 2024, further reducing access to care [8]. These barriers to care can intensify psychological distress and create a syndemic environment that significantly increases the risk of poor mental health for TGD people [6].

Gender-affirming hormone therapy (GAHT), which involves medications such as testosterone, estrogens, and antiandrogens [9], has been associated with improvements in depression, anxiety, suicidality, and overall quality of life for transgender people [4-6]. A 2022 randomized controlled trial (RCT) conducted in Australia found a significant reduction in depression and suicidality within 3 months for patients beginning testosterone therapy compared to those for whom access to care was delayed [10]. However, the study may not represent individuals using estrogen or those outside of Australia, where care barriers differ from those in the United States. For example, TGD individuals in the United States face varied access challenges [10-12] particularly in states [10] where recent legislation led to legal penalties for providers of gender-affirming care [13], clinic closures, and care disruptions. These laws profoundly impact TGD individuals’ mental health [14,15]. Studies that include a nationwide population of TGD adults can help ensure that the findings are applicable across the United States.

Further, the rapid expansion of telehealth and acceptance of telehealth-delivered GAHT [7] after COVID-19 has shifted the way this care is provided. Telehealth is a critical modality for primary care [8], mental [16], and behavioral health [15], yet its efficacy in delivering GAHT remains less explored [11,17]. TGD adults report frequent use of telemedicine [10], and express high levels of satisfaction with telehealth-delivered GAHT [12]. Therefore, understanding changes in health outcomes among those initiating GAHT via telehealth is essential to ensure that this increasingly widespread model of care provides effective, equitable, and accessible treatment.

Factors that are associated with improved mental health outcomes for those initiating GAHT are not well understood. Previous research found the mental health of TGD individuals varies based on insurance coverage [13], legal affirmation of gender [14,18], gender-affirming surgery access [19], geography [20], gender identity [21], race and ethnicity [9], employment status, and mental health treatment [22]. Few studies have explored the association between this comprehensive set of factors and changes in mental health outcomes.

We conducted an observational study of adults in 43 states initiating GAHT for the first time at a telehealth clinic to assess changes in depression, anxiety, and suicide ideation over a 3-month period. In addition, we examined whether these changes varied based on demographic factors, insurance coverage, and mental health treatment.

## Methods

### Overview

#### Ethical Considerations

This study received an exemption from the Western Institutional Review Board-Copernicus Group (WCG) institutional review board. The study follows the Strengthening the Reporting of Observational Studies in Epidemiology (STROBE) reporting guideline.

#### Study Procedures (Participant Selection and Study Framework)

We conducted a single-arm, observational study at FOLX Health, a telehealth clinic licensed across all 50 US states and the District of Columbia, with adults aged 18 years and older who initiated testosterone or estrogen therapy between August 3, 2023, and November 6, 2023. Participants were required to complete an intake questionnaire before their initial visit and a follow-up assessment 3-4 months later. All had consented to care and were first-time GAHT recipients, and the study did not influence their treatment outcomes.

All participants self-scheduled their initial GAHT visit and completed a comprehensive intake questionnaire before the visit, and a follow-up questionnaire three months later to assess mental health and care satisfaction. Alerts were sent if urgent mental health issues were reported. Initial Zoom visits lasted 30 minutes, with payment options including insurance or cash, and prescriptions could be sent to local pharmacies or delivered directly to homes. Treatment options, such as injectable testosterone cypionate or enanthate, transdermal testosterone gel, estradiol tablets, injectable estradiol cypionate or valerate, and transdermal estrogen patches, were chosen based on standard care practices and patient goals, under clinician guidance [23]. Psychotherapy was not a requirement for initiation of GAHT [23].

### Measures

#### Psychological Outcomes

The primary outcomes of this study were the resolution of suicide ideation, and Healthcare Effectiveness Data and Information Set measures for response and remission from depression and anxiety [24], assessed using the Patient Health Questionnaire-9 (PHQ-9) [25] for depression and the General Anxiety Disorder-7 (GAD-7) for anxiety [24,26]. Elevated symptoms were defined as PHQ-9 ≥10 and GAD-7 ≥8 [27,28]. Response, our primary outcome, was defined as a 50% reduction in these scores [29,30], accommodating patients with various baseline severities [29]. Remission, chosen as a secondary outcome for its correlation with improved daily function and long-term prognosis, was defined as scores below 5 on both tools. Completion of the PHQ-9 and GAD-7 was mandatory at baseline, ensuring that all respondents provided data for these instruments.

Suicidal ideation was evaluated with item 9 of the PHQ-9, “Thoughts that you would be better off dead or of hurting yourself in some way.” Consistent with existing research and the relevance of this measure for gender-diverse populations [2,31], any response other than “not at all” indicated possible suicidal ideation. Resolution was defined as a “not at all” response at the follow-up (refer to Multimedia Appendix 1).

#### Covariates Plausibly Associated With Changes in Outcomes

We included demographic factors (age, sex assigned at birth, race or ethnicity, and legal change of gender marker on identity document), access factors (insurance coverage, urbanicity of living environment, educational level, and unemployment status), and health care factors (history of a gender-affirming surgery (GAS); use of mental health treatment, including medication for anxiety or depression; use of talk therapy in the year previous [32] to initiating hormones; and use of talk therapy during the study period).

#### Statistical Analysis

First, we present baseline descriptive statistics, categorizing continuous variables as mean (SD) and categorical variables as frequency (percentage). These statistics are provided separately for individuals seeking testosterone and estrogen therapy.

Second, we calculated the mean changes in PHQ-9 and GAD-7 scores for all participants, along with their 95% CIs. Next, we analyzed the proportion of individuals achieving response and remission from depression and anxiety, and resolution of suicide ideation.

Third, for each group —those with baseline elevated symptoms of depression (PHQ-9 ≥10) anxiety (GAD-7 ≥8), and suicide ideation— we estimated 5 separate models for each outcome: resolution of suicide ideation, and response and remission of anxiety and depression. We used generalized estimating equations with a binomial family and a logit link function to estimate adjusted odds ratios for outcomes while accounting for individual-level clustering. These models estimated the association between outcomes and any mental health treatment, health insurance, education, urbanicity of residence, age, and sex assigned at birth. All models included time to follow-up (in weeks), and because remission does not take into account baseline symptom severity, we also included baseline PHQ-9 and GAD-7 scores in those models. We excluded covariates with a prevalence of <5% and state of residence from the final model to prevent overfitting and ensure the reliability of our estimates.

Participants with missing data on PHQ-9 or GAD-7 scores at baseline or follow-up were excluded from the analysis. For all other variables, missing data were categorized separately and included in the analysis to maintain the integrity and size of the sample.

We conducted a sensitivity analysis in which we reran all models after excluding individuals who had experienced mental health treatment, as well as those who had undergone GAS or legally changed their gender. This approach aimed to help mitigate the effects of unobserved confounders like symptom persistence and perceived need for care, which could influence mental health independently.

Finally, to assess potential differences between participants who completed the follow-up survey and those who did not, we analyzed the association of completing the follow-up survey with baseline PHQ-9 and GAD-7 scores, controlling for time and covariates described previously.

Statistical analyses were conducted using Python (version 3.11.7; Python Software Foundation) in March 2024.

## Results

### Overview

A total of 669 adults initiated GAHT via telehealth during the dates specified in our study period, with 98% (658/669) completing an initial intake survey; and 52% (342/669) participating in a follow-up survey. A detailed comparison between participants who completed the follow-up and those who did not is available in Multimedia Appendix 2. There were no baseline mental health differences between participants who did and did not complete the follow-up survey.

Our final sample included 342 adults ages 18 years to 67 years (mean 26.5 years [SD 8.5 years]). Among 342 adults, 192 (56.1%) were prescribed estrogen therapy and 150 (43.9%) were prescribed testosterone therapy.

Prescriptions were issued primarily by nurse practitioners (291/342, 85%), and the remainder were primary care physicians (51/342, 15%). Most patients (298/342, 87%) completed their first appointment within 7 days of registration. Prescription fulfillment was split, with 50% (171/342) receiving medications through direct mail and 50% (171/342) through local pharmacies. Estradiol tablets were the predominant form of estrogen therapy, while injectable testosterone cypionate was the most common testosterone therapy (Multimedia Appendix 3).

Overall, 71% (243/342) identified their race as white only, and 12% (42/342) identified their ethnicity as Latinx (Table 1). Geographic distribution showed a concentration in the southern United States (170/342, 50%), and socioeconomic data revealed that 59% (201/342) were uninsured and 6% (18/342) unemployed, with educational attainment distributed as follows: 36% (122/342) with high school education or less, 37% (124/342) with some college education, and 27% (93/342) holding at least a college degree.

**Table 1:** Baseline characteristics of adults initiating gender-affirming hormone therapy through telehealth.

		All (n=342)	Estrogen (n=192)	Testosterone (n=150)	*P* value^a^
**Age group, n (%)**					
	18-21	122 (35.67)	56 (29.17)	66 (44)	.04
	22-26	84 (24.56)	51 (26.56)	33 (22)	
	27-31	68 (19.88)	41 (21.35)	27 (18)	
	≥32	68 (19.88)	44 (22.92)	24 (16)	
**Region^b^, n (%)**					
	Midwest	43 (12.57)	29 (15.1)	14 (9.33)	.08
	North	54 (15.79)	36 (18.75)	18 (12)	
	South	170 (49.71)	90 (46.88)	80 (53.33)	
	West	75 (21.93)	37 (19.27)	38 (25.33)	
**Urbanicity, n (%)**					
	City	75 (21.99)	36 (18.75)	39 (26.17)	.35
	Suburb	112 (32.84)	68 (35.42)	44 (29.53)	
	Small town	123 (36.07)	69 (35.94)	54 (36.24)	
	Rural	31 (9.09)	19 (9.9)	12 (8.05)	
	Missing	1 (0.3)	0 (0)	1 (0.02)	
**White alone, n (%)**					
	Yes	243 (71.47)	142 (74.74)	101 (67.33)	.17
	No	97 (28.53)	48 (25.26)	49 (32.67)	
	Missing	2 (0.58)	2 (0.1)	0 (0)	
**Latinx, n (%)**					
	Yes	42 (12.28)	18 (9.38)	24 (16)	.09
	No	300 (87.72)	174 (90.62)	126 (84)	
**Changed legal gender, n (%)**					
	Yes	16 (4.68)	5 (2.6)	11 (7.33)	.07
	No	326 (95.32)	187 (97.4)	139 (92.67)	
**Gender identity, n (%)**					
	Woman or trans woman	121 (35.48)	118 (61.78)	3 (2)	<.001
	Man or trans man	108 (31.67)	11 (5.76)	97 (64.67)	
	Nonbinary	99 (29.03)	55 (28.8)	44 (29.33)	
	Other	13 (3.81)	7 (3.66)	6 (4)	
	Missing	1 (0.29)	1 (0.52)	0	
**Education category, n (%)**					
	High school or less	122 (35.99)	65 (34.21)	57 (38.26)	.69
	Some college	124 (36.58)	70 (36.84)	54 (36.24)	
	College graduate or higher	93 (27.43)	55 (28.95)	38 (25.5)	
**Unemployment status, n (%)**					
	Yes	18 (5.57)	5 (2.76)	13 (9.15)	.03
	No	305 (94.43)	176 (97.24)	129 (90.85)	
	Missing	19 (5.53)	11 (5.72)	8 (5.33)	
**Insurance type, n (%)**					
	In-network	101 (29.53)	50 (26.04)	51 (34)	.13
	Out-of-network	40 (11.7)	20 (10.42)	20 (13.33)	
	Uninsured	201 (58.77)	122 (63.54)	79 (52.67)	
**Pharmacy type, n (%)**					
	Local pharmacy	170 (49.71)	95 (49.48)	75 (50)	1.00
	Shipped to home	172 (50.29)	97 (50.52)	75 (50)	
**Had a mental health visit in past year, n (%)**					
	Yes	132 (38.6)	63 (32.81)	69 (46)	.02
	No	210 (61.4)	129 (67.19)	81 (54)	
Number of mental health visits in previous year, mean (SD)		6.01 (12.51)	4.62 (10.64)	7.78 (14.39)	.03
**Suicide ideation at baseline, n (%)**					
	Yes	85 (24.85)	52 (27.08)	33 (22)	.34
	No	257 (75.15)	140 (72.92)	117 (78)	
**Baseline PHQ-9^c^ category, n (%)**					
	None or minimal: 0-4	128 (37.43)	72 (37.5)	56 (37.33)	.38
	Mild: 5-9	78 (22.81)	44 (22.92)	34 (22.67)	
	Moderate: 10-14	76 (22.22)	37 (19.27)	39 (26)	
	^d^Moderately Severe: 15-19	42 (12.28)	26 (13.54)	16 (10.67)	
	^d^Severe: 20-27	18 (5.26)	13 (6.77)	5 (3.33)	
**Baseline GAD-7^e^category, n (%)**					
	None/Minimal: 0-4	127 (37.13)	73 (38.02)	54 (36)	.91
	Mild: 5-7	91 (26.61)	51 (26.56)	40 (26.67)	
	^f^Mild: 8-9	40 (11.7)	20 (10.42)	20 (13.33)	
	^f^Moderately Severe: 10-14	51 (14.91)	28 (14.58)	23 (15.33)	
	^f^Severe: 15-21	33 (9.65)	20 (10.42)	13 (8.67)	
**Prescribed a medication for anxiety or depression, n (%)**					
	Yes	82 (23.98)	36 (18.75)	46 (30.67)	.02
	No	260 (76.02)	156 (81.25)	104 (69.33)	
**Had gender-affirming surgery, n (%)**					
	Yes	14 (4.09)		14 (9.33)	<.001
	No	328 (95.91)	192 (100)	136 (90.67)	

^a^*P* values represent the statistical significance of differences between adults prescribed testosterone and estrogen, calculated using appropriate tests for each variable type (eg, chi-square test for categorical variables and *t* test for continuous variables).

^b^Patients resided in 43 states.

^c^PHQ-9: Patient Health Questionnaire-9.

^d^Probable major depression.

^e^GAD-7: General Anxiety Disorder-7

^f^Probably anxiety disorder.

Only 5% (16/342) of participants had legally changed their gender on their license or identification card, and 4% (14/342) had undergone GAS. Meanwhile, 39% (132/342) had consulted a mental health professional in the past year, with an average of six visits, and 24% (82/342) were prescribed medication for anxiety or depression.

Individuals seeking testosterone therapy were younger, used more mental health care services, had higher rates of prescriptions for anxiety or depression, and were more likely to have undergone one or more GAS compared to those seeking estrogen therapy.

### Baseline and Follow-Up Depression and Anxiety Scores

**Figure 1:**
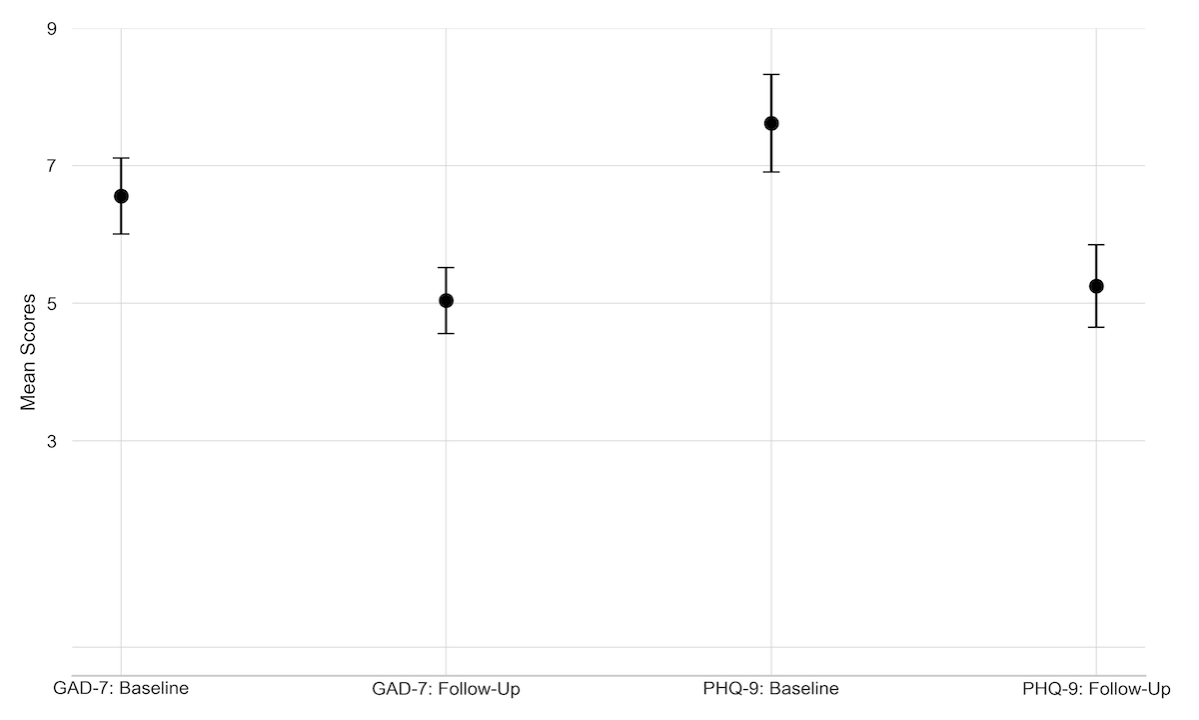
Baseline and follow-up depression (PHQ-9) and anxiety (GAD-7) scores among adults who initiated gender-affirming hormone therapy. GAD-7: General Anxiety Disorder-7; PHQ-9: Patient Health Questionnaire-9.

There were 333 individuals who reported depression and anxiety scores at both baseline and follow-up. The mean baseline PHQ-9 score was 7.6 (Figure 1). At follow-up, there was a statistically significant decrease in PHQ-9 scores (mean difference, −2.4 points; 95% CI −3.0 to −1.8 points).

Mean and 95% CIs are represented for 333 adults who completed baseline and follow-up PHQ-9 and GAD-7 assessments.

In total, 40% (136/342) had elevated symptoms of depression (PHQ-9 ≥10) at baseline. This included 76 individuals who scored moderate (10 to 14), 42 individuals who scored moderately severe (15 to 19), and 18 individuals who scored severe (20 to 27). For those without elevated symptoms, the baseline PHQ-9 score was none/minimal (0 to 4) in 128 individuals (128/342, 37%), and mild (5 to 9) in 78 individuals (78/342, 23%).

The mean baseline GAD-7 score was 6.6. At follow-up, there was a statistically significant decrease in GAD-7 scores (mean difference −1.5 points, 95% CI −2.0 to −1.0 points).

In total, 36% (120/342) had elevated symptoms of anxiety (GAD-7 ≥8). This included 40 individuals who scored mild (8-9), 51 individuals who scored moderately severe (10 to 14), and 33 individuals who scored severe (15 to 21). For those without elevated symptoms, the baseline GAD-7 score was none or minimal (0 to 4) in 127 individuals (127/342, 37%), and mild (5 to 7) in 91 individuals (91/342, 27%).

### Response and Remission from Depression and Anxiety

**Figure 2:**
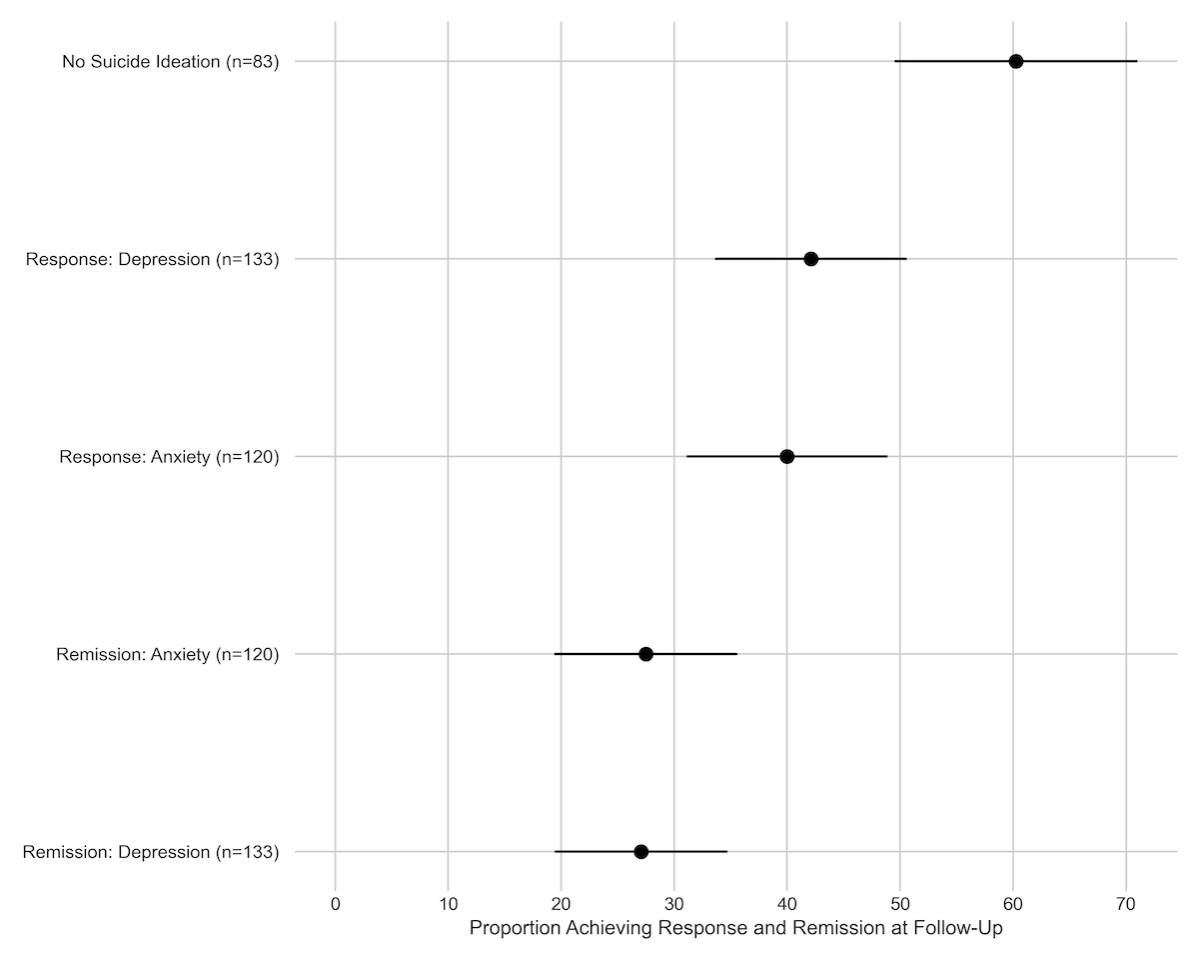
The proportion of adults who initiated GAHT (gender-affirming hormone therapy) achieving remission and response from elevated symptoms of depression and anxiety, and resolution of suicide ideation at follow-up.

Among the 133 individuals with PHQ-9 ≥10 at baseline, 42% (n=56) achieved response (50% reduction in score), and 27% (n=36) achieved remission (PHQ-9 <5) at follow-up (Figure 2).

Among the 120 individuals with GAD-7 ≥8 at baseline, 40% (n=48) achieved response, and 28% (n=33) achieved remission at follow-up.

### Resolution of Suicide Ideation

At baseline, 83 individuals (83/342, 25%) reported suicide ideation. Among these individuals, 60% (50/83) did not report suicide ideation at follow-up.

### Adjusted Odds of Response and Remission

Use of mental health treatment before or during the period was associated with a 0.43 (95% CI 0.19-0.93) lower odds of depression response and 0.43 (95% CI 0.19-0.98) lower odds of anxiety response. Those who were female sex assigned at birth (FSAB) had a 0.38 (95% CI 0.18-0.82) lower odds of depression response. No statistically significant association was found between depression or anxiety symptom response and age, race or ethnicity, insurance coverage, educational level, or use of mental health treatment (Table 2).

No associations were observed between mental health treatment or other baseline factors and remission of depression, anxiety, and resolution of suicide ideation (Table 3).

**Table 2:** Association between demographic and treatment factors and mental health response among adults initiating gender-affirming hormone therapy with elevated symptoms of depression or anxiety.

			Response from depression (n= 133)^a^					Response from anxiety (n= 120)^b^		
			Adjusted odds ratio (95% CI)		*P* value		Adjusted odds ratio (95% CI)			*P* value
**Any mental health treatment^c^**			0.43 (0.19-0.93)		.03		0.43 (0.19-0.98)			.04
**Age (in years)**			1.06 (0.99-1.13)		.10		1.03 (0.97-1.11)			.34
**Sex assigned at birth**										
	Male sex assigned at birth	1 (Reference)					1 (Reference)			
	Female sex assigned at birth	0.38 (0.18-0.82)		.01		0.77 (0.34-1.73)			.52	
	Non-Latinx White alone	1.66 (0.73-3.75)		.22		0.77 (0.32-1.83)			.55	
**Insurance status**										
	Uninsured	1 (Reference)					1 (Reference)			
	Insured	0.64 (0.29-1.43)		.27		0.81 (0.37-1.79)			.61	
**Urbanicity of residence**										
	City or suburb	1 (Reference)					1 (Reference)			
	Rural or small town	1.22 (0.56-2.63)		.62		1.36 (0.62-3.02)			.44	
**Education**										
	Some college or higher	1 (Reference)					1 (Reference)			
	High school or less	0.83 (0.38-1.82)		.63		0.86 (0.36-2.02)			.73	

^a^All models use Generalized Estimating Equations with a binomial family and a logit link function, with clustering at the individual level and controlling for time to follow-up. We excluded employment status, change of legal gender, or history of a gender-affirming surgery because of small cell sizes. This model includes those with a Patient Health Questionnaire-9 score ≥10 at baseline. The response is defined as a ≥50% improvement in the Patient Health Questionnaire-9 score from baseline.

^b^Model includes those with a General Anxiety Disorder-7 score ≥8 at baseline. The response is defined as a ≥50% improvement in the General Anxiety Disorder-7 score.

^c^Any mental health treatment was defined as using an antidepressant or talk therapy a year before or during the study period.

**Table 3:** Association between demographic and treatment factors and mental health remission among adults initiating gender-affirming hormone therapy.

		Remission from depression^a^ (n=133)			Remission from anxiety^b^ (n=120)			Remission from suicide ideation^c^ (n=83)	
		Adjusted odds ratio (95% CI)	*P* value	Adjusted odds ratio (95% CI)		*P* value	Adjusted odds ratio (95% CI)		*P* value
Any mental health treatment^d^		0.42 (0.17-1.03)	.06	0.49 (0.19-1.26)		.14	2.46 (0.87-6.99)		.09
Age (in years)		1.06 (0.99-1.14)	.11	1.01 (0.94-1.08)		.84	1.01 (0.91-1.11)		.86
**Sex assigned at birth**									
	Male	1 (Reference)			1 (Reference)			1 (Reference)	
	Female	0.52 (0.21-1.25)	.14	0.58 (0.25-1.38)		.22	0.43 (0.14-1.30)		.14
	Non-Latinx White alone	2.01 (0.82-4.92)	.13	0.75 (0.28-1.98)		.56	0.75 (0.27-2.09)		.58
**Insurance status**									
	Uninsured	1 (Reference)			1 (Reference)			1 (Reference)	
	Insured	0.55 (0.21-1.44)	.22	0.76 (0.31-1.88)		.55	0.46 (0.17-1.26)		.13
**Urbanicity of residence**									
	City or suburb	1 (Reference)			1 (Reference)			1 (Reference)	
	Rural or small town	1.53 (0.64-3.62)	.34	1.61 (0.68-3.81)		.28	1.17 (0.43-3.21)		.76
**Education**									
	Some college or higher	1 (Reference)			1 (Reference)			1 (Reference)	
	High school or less	1.25 (0.54-2.93)	.61	0.53 (0.20-1.43)		.21	1.40 (0.48-4.09)		.53
	GAD-7^e^ or PHQ-9^f^ baseline	0.93 (0.84-1.03)	.14	0.91 (0.80-1.04)		.16			

^a^All models use Generalized Estimating Equations with a binomial family and a logit link function, with clustering at the individual level and controlling for time to follow-up. We excluded employment status, change of legal gender, or history of a gender-affirming surgery because of small cell sizes. This model includes those with PHQ-9 ≥10 at baseline. Remission is defined as a PHQ-9 <5 at follow-up

^b^Model includes those with GAD-7 ≥8 at baseline. Remission is defined as a GAD-7 score <4 at follow-up.

^c^Model includes those with suicide ideation at baseline. Remission includes those who do not have suicide ideation at follow-up.

^d^Any mental health treatment was defined as using an antidepressant or using talk therapy a year before or during the study period.

^e^GAD-7: General Anxiety Disorder-7.

^f^PHQ-9: Patient Health Questionnaire-9.

### Sensitivity Analyses

After excluding those without mental health treatment, GAS, or a legal gender change, no statistically significant association was observed between FSAB and odds of depression and response or remission (Multimedia Appendices 4 and 5).

## Discussion

### Principal Findings

Among adults newly initiating testosterone or estrogen therapy via telehealth services with elevated depression and anxiety symptoms, 40%-42% achieved a clinically meaningful response. Of those with suicide ideation, 60% saw resolution within 3 months. These results are consistent with improvements reported in previous studies [4,5,33]; however, most of these studies were conducted outside the United States, inclusive of youth, and before the COVID-19 pandemic, thus telehealth modalities were not specifically explored. Moreover, very few studies have used similar outcome measures. One such example is an observational study of 315 youth aged 13 to 20 from Seattle. This study reported no change in moderate to severe depressive symptoms at 3 or 6 months, with improvements only evident after a year on GAHT [34].

Other US-based studies, including smaller cohorts from New York, California, Boston, and Illinois, also documented mental health improvements in youth initiating GAHT using other measures [6,35]. While comparisons between observational studies and RCTs, may raise concerns due to differences in study design, it is noteworthy that 61% of Australian adults receiving testosterone saw a reduction of five or more points on the PHQ-9 score, and 52% experienced the resolution of suicide ideation within three months [34]. Our study fills a critical gap by providing contemporary insights on hormone therapy via telehealth, demonstrating significant mental health improvements in a setting largely unexplored in previous research.

Although GAHT is not explicitly prescribed for anxiety or depression, its impact on mental health can be profound, and in this study, it compares favorably with treatments that target mental health conditions directly. For instance, antidepressants typically show an improvement in symptoms for less than 20% of adults with moderate to severe depression [36,37]. Other types of treatments show similar effects, with 13-25% of adults achieving remission from depression in traditional and telehealth-enabled collaborative care models [38,39]. While GAHT is not a replacement for mental health condition–specific treatments, this research strongly suggests that it is an important factor in mental health wellness for TGD adults.

Our finding that individuals who used mental health treatment had lower odds of achieving a depression response, though not remission, suggests that those with a history of mental health treatment may have more persistent symptoms of depression. In contrast, individuals without prior treatment may have more emergent symptoms. Furthermore, engagement in mental health treatment is not randomly assigned; rather, it is heavily influenced by both perceived needs and available resources [36]. Notably, less than 40% of this population had health insurance, which likely limited their access to consistent and comprehensive mental health treatment. Our findings imply that improvements in mental health observed in individuals using hormone therapy occurred independently of their use of other mental health treatments.

Prior studies have reported that adults with multiple marginalized identities are at risk for worse mental health as a result of compounded discrimination [21]. While our findings showed no significant differences in mental health remission based on race, ethnicity, or education, we observed that FSAB had lower odds of depression response than male sex assigned at birth adults. However, this association disappeared when excluding individuals already receiving mental health treatment. The higher rates of mental health treatment among FSAB individuals suggest variations in underlying symptomatology, which our main models could not fully adjust for. Future research should further elucidate how concurrent GAHT and mental health treatment influence mental health symptoms.

### Limitations

A primary limitation is the lack of a control group of participants who did not receive GAHT, which would be both infeasible and unethical. This makes our observational study unable to definitively link improvements in mental health to GAHT alone due to potential unmeasured factors. In addition, with only a 3-month follow-up, the long-term effects of GAHT remain unclear. Our reliance on patient-reported outcomes may introduce bias, although baseline mental health was comparable between those lost to follow-up and those retained. Furthermore, the quick scheduling of initial visits within a week, contrasting with the months- to years-long delays at traditional gender specialty clinics, may also influence the generalizability of our results.

The generalizability of our findings may be limited to similar care models, as our cohort was drawn from a specialized telehealth-only clinic—one of a few in the United States providing GAHT. Although telehealth has increasingly been recognized as a way to broaden geographic access to such care [7,40], it remains out of reach for some populations. Compared to other nationally representative samples of transgender and gender-diverse adults, our participants were generally younger, more educated, predominantly white, more rural, and less insured [1,41] Despite these differences, the prevalence of mental health symptoms before GAHT mirror those of other clinics providing in-person care, suggesting that our results may be comparable with other health care settings [2,27].

### Conclusions

In this observational study, adults accessing GAHT at a telehealth clinic experienced significant improvements in depression, anxiety, and suicide ideation after 3 months. Although GAHT is not prescribed for mental health, it may be an important part of mental health wellness for TGD adults. Future studies with longer follow-up periods are essential to better understand these findings.

## Appendix

Multimedia Appendix 1 Contains detailed description of measures used in the study.

Multimedia Appendix 2 Differences in baseline characteristics of participants and nonparticipants in the follow-up assessment.

Multimedia Appendix 3 Proportion of study participants by type of initial prescription of estrogen or testosterone.

Multimedia Appendix 4 Sensitivity analysis: association between demographic factors and mental health response among those without mental health treatment, gender-affirming surgery, or legal gender change.

Multimedia Appendix 5 Sensitivity analysis: association between demographic and mental health remission among those without mental health treatment, gender-affirming surgery, or legal gender change.

## Abbreviations

FSAB: female sex assigned at birth
GAD-7: General Anxiety Disorder-7
GAHT: gender-affirming hormone therapy
GAS: gender-affirming surgery
PHQ-9: Patient Health Questionnaire-9
RCT: randomized controlled trial
STROBE: Reporting of Observational Studies in Epidemiology
TGD: transgender and gender diverse
WCG: WIRB-Copernicus Group
