# Supplementary material for: Mental Health Changes in US Transgender Adults Beginning Hormone Therapy Via Telehealth: Longitudinal Cohort Study
Source: J Med Internet Res. 2025 Feb 14;27:e64017. doi: 10.2196/64017 (PMC11888058; doi:10.2196/64017)
Supplement: Multimedia Appendix 1 [file jmir_v27i1e64017_app1.pdf]

Multimedia Table1. Survey Instruments

Mental Health Outcome Measures	
Generalized Anxiety Disorder 7-item scale (GAD-7)	*Over the last 2 weeks, how often have you been bothered by the following problems?*
	1. Feeling nervous, anxious, or on edge
	2. Not being able to stop or control worrying
	3. Worrying to much about different things
	4. Trouble relaxing
	5. Being so restless that it's hard to sit still
	6. Becoming easily annoyed or irritable
	7. Feeling afraid as if something awful might happen
Patient Health Questionnaire 9-item scale (PHQ-9) for Depression	*Over the past 2 weeks, how often have you been bothered by any of the following problems?*
	1. Little interest or pleasure in doing things
	2. Feeling down, depressed or hopeless
	3. Trouble falling asleep, staying asleep, or sleeping too much
	4. Feeling tired or having little energy
	5. Poor appetite or overeating
	6. Feeling bad about yourself – or that you're a failure or have let yourself or your family down.
	7. Trouble concentrating on things, such as reading the newspaper or watching television
	8. Moving or speaking so slowly that other people could have noticed. Or, the opposite –
	being so fidgety or restless that you have been moving around a lot more than usual.
	9. Thoughts that you would be better off dead or of hurting yourself in some way
Suicide Ideation	"Over the past 2 weeks, how often have you been bothered by thoughts that you would be better off dead or of hurting yourself in some way?" (Item-9 from the PHQ-9)
**Healthcare Factors**	
Baseline Mental Health Utilization	*In the last 12 months, how many visits have you had to a mental health provider like a therapist?*

Follow-Up Mental Health Utilization	*Since your first visit with our clinic, how many visits have you had to a mental health provider like a therapist?*
Mental Health Prescriptions	alprazolam, amitriptyline, amoxapine, asendin, ativan, bupropion, buspar, buspirone, celexa, citalopram, cymbalta, desipramine, desvenlafaxine, desyrel, doxepin, duloxetine, effexor, elavil, escitalopram, fetzima, fluoxetine, imipramine, khedezla, levomilnacipran, lexapro, lorazepam, ludiomil, maprotiline, nefazodone, norpramin, nortriptyline, pamelor, paroxetine, paxil, pexeva, pristiq, protriptyline, prozac, sertraline, serzone, sinequan, surmontil, tofranil, trazodone, trimipramine, trintellix, venlafaxine, viibryd, vilazodone, vivactil, vortioxetine, wellbutrin, xanax, zoloft
History of Gender Affirming Surgery	*Select all surgeries you have had.* Reported at least one of the following: Top Surgery - Breast Augmentation / Implants, Top Surgery - Chest Reduction / Mastectomy, Gender affirming genital surgery, Oophorectomy / Removal of Ovaries, or Hysterectomy
**Demographic Factors**	
Age	Age at visit was calculated from the birth date provided at registration and the visit date.
Sex Assigned at Birth	Individuals initiating testosterone therapy were assigned female at birth, while those starting estrogen therapy were assigned male at birth.
Gender Identity	*If you had to choose only one of the following terms, which best describes your current gender identity?* Responses: Woman / Trans Woman, Man / Trans Man, Non-binary / Genderqueer / Gender Diverse / Two-Spirit (2S), Gender Not Listed Here / Other
Race / Ethnicity	*What is your race/ethnicity? (Please select all that apply)* Responses: White, Black or African American, Asian, Hispanic or Latino, Native American or Indigenous, Pacific Islander, Mixed race/ethnicity, Other
Legal Change of Gender	Individuals were considered to have legally changed their gender if their legal gender on their license or identification card at registration differed from their sex assigned at birth.
**Access Factors**	
Insurance Coverage	Individuals were assigned to be In-Network, Out-of-Network, or Uninsured based on their payment method for first visit.
Region	Individuals were assigned to a U.S. Census Region based on their state of residence at registration.
Urbanicity of Living Environment	*How would you describe the place where you live?* Responses: Large city, Suburb near a large city, Small city or town, Rural area
Educational Attainment	*What is the highest level of education you have completed?* Responses: High school graduate or less, Some college, College graduate or higher
Unemployment Status	*What is your current employment status?* Responses: Unemployed but looking for work or Unemployed but stopped looking for work
