# Supplementary material for: Mental Health Changes in US Transgender Adults Beginning Hormone Therapy Via Telehealth: Longitudinal Cohort Study
Source: J Med Internet Res. 2025 Feb 14;27:e64017. doi: 10.2196/64017 (PMC11888058; doi:10.2196/64017)
Supplement: Multimedia Appendix 2 [file jmir_v27i1e64017_app2.pdf]

Multimedia Table 2. Differences in Baseline Characteristics of Participants and Non-Participants in the Follow-Up Assessment

		No Follow-Up	Follow-Up	*P*-Value[a]
n		316	342	
Suicide Ideation at Baseline, mean (SD)		0.23 (0.42)	0.25 (0.43)	0.53
Moderate or Severe Depression[b], mean (SD)		0.33 (0.47)	0.40 (0.49)	0.07
Moderate or Severe Anxiety[c], mean (SD)		0.36 (0.48)	0.36 (0.48)	0.88
GAHT Type, n (%)	Estrogen	199 (64.19)	192 (56.14)	0.04
	Testosterone	111 (35.81)	150 (43.86)	
Age Group, n (%)	18-21	120 (38.83)	122 (35.67)	0.02
	22-26	98 (31.72)	84 (24.56)	
	27-31	39 (12.62)	68 (19.88)	
	32+	52 (16.83)	68 (19.88)	
Region, n (%)	M	42 (13.59)	43 (12.57)	0.88
	N	45 (14.56)	54 (15.79)	
	S	148 (47.90)	170 (49.71)	
	W	74 (23.95)	75 (21.93)	
Urbanicity, n (%)	City	82 (26.28)	75 (21.99)	0.26
	Rural	18 (5.77)	31 (9.09)	
	Small town	105 (33.65)	123 (36.07)	
	Suburb	107 (34.29)	112 (32.84)	
White Alone, n (%)	No	108 (34.84)	97 (28.53)	0.10
	Yes	202 (65.16)	243 (71.47)	
Latinx, n (%)	No	267 (84.49)	300 (87.72)	0.28
	Yes	49 (15.51)	42 (12.28)	
Gender Identity, n (%)	Man / Trans Man	80 (25.72)	108 (31.67)	0.19
	Non-binary	91 (29.26)	99 (29.03)	
	Other	8 (2.57)	13 (3.81)	
	Woman / Trans Woman	132 (42.44)	121 (35.48)	

Educational Attainment, n (%)	High School or Less	122 (38.61)	122 (35.67)	0.27
	Some college	111 (35.13)	124 (36.26)	
	College graduate or higher	75 (23.73)	93 (27.19)	
	Missing	8 (2.53)	3 (0.88)	
Had a mental health visit in past year, n (%)	No	183 (57.91)	210 (61.40)	0.41
	Yes	133 (42.09)	132 (38.60)	
Insurance Type, n (%)	In-Network	87 (28.16)	101 (29.53)	0.69
	Out-of-Network	43 (13.92)	40 (11.70)	
	Uninsured	179 (57.93)	201 (58.77)	

[a] *P*-Values represent the statistical significance of differences between those who didn't complete the follow-up survey and those who did complete it and were part of the final cohort, calculated using appropriate tests for each variable type (e.g., chi-squared test for categorical variables and t-test for continuous variables).
[b] Patient Health Questionnaire 9-item score >=10
[c] Generalized Anxiety Disorder 7-item>=8
Abbreviations: GAHT; Gender Affirming Hormone Therapy
