# Supplementary material for: Mental Health Changes in US Transgender Adults Beginning Hormone Therapy Via Telehealth: Longitudinal Cohort Study
Source: J Med Internet Res. 2025 Feb 14;27:e64017. doi: 10.2196/64017 (PMC11888058; doi:10.2196/64017)
Supplement: Multimedia Appendix 3 [file jmir_v27i1e64017_app3.pdf]

Multimedia Table 3. Initial Prescription of Estrogen or Testosterone Formulations

Hormone Type	Primary Prescription	Individuals As % of Type
Estrogen	estradiol 1 or 2 mg tablet	73.1%
Estrogen	estradiol valerate 20 or 40 mg/mL intramuscular oil	21.8%
Estrogen	estradiol 0.1 mg/24 hr semiweekly transdermal patch	5.2%
Testosterone	testosterone cypionate 200 mg/mL intramuscular oil	75.5%
Testosterone	testosterone 12.5 mg or 20.25 mg/1.25 gram per pump act.(1.62 %) transdermal gel	24.5%
