# Supplementary material for: Mental Health Changes in US Transgender Adults Beginning Hormone Therapy Via Telehealth: Longitudinal Cohort Study
Source: J Med Internet Res. 2025 Feb 14;27:e64017. doi: 10.2196/64017 (PMC11888058; doi:10.2196/64017)
Supplement: Multimedia Appendix 4 [file jmir_v27i1e64017_app4.pdf]

Multimedia Table 4. Sensitivity Analysis: Association Between Demographic Factors and Mental Health Response Among Those Without Mental Health Treatment,[c] GAS, or Legal Gender Change

	Response from Moderate or Severe Depression (n=71)[a]			Response from Moderate or Severe Anxiety (n=63)[b]	
	aOR (95% CI)	*P* value		aOR (95% CI)	*P* value
Age (in years)	1.08 (0.95, 1.21)	0.24		1.06 (0.95, 1.19)	0.32
Sex Assigned at Birth					
Male	1 [Reference]			1 [Reference]	
Female	0.22 (0.16, 1.21)	0.08		0.38 (0.11, 1.35)	0.14
Non-Latinx White Alone	0.76 (0.25, 2.28)	0.62		0.46 (0.14, 1.50)	0.20
Insurance Status					
Uninsured	1 [Reference]			1 [Reference]	
Insured	0.51 (0.16, 1.63)	0.25		0.75 (0.24, 2.32)	0.62
Urbanicity of Residence					
City or Suburb	1 [Reference]			1 [Reference]	
Rural or Small Town	1.83 (0.58, 5.84)	0.31		1.83 (0.53, 6.39)	0.34
Education					
Some College or Higher	1 [Reference]			1 [Reference]	
High school or less	0.61 (0.22, 1.70)	0.35		0.69 (0.24, 2.01)	0.50

[a]All models use Generalized Estimating Equations (GEE) with a binomial family and a logit link function, with clustering at the individual level and controlling for time to follow-up. This model includes those with PHQ-9>=10 at baseline. Response is defined as a >=50% improvement in PHQ score from baseline.
[b] Model includes those with GAD-7>=8 at baseline.Response is defined as a >=50% improvement in GAD score from baseline.
[c] Any mental health treatment was defined as using an antidepressant, or using talk therapy a year before or during the study period.
Abbreviations: GAS, Gender-Affirming Surgery, GAD-7, Generalized Anxiety Disorder 7-item scale; PHQ-9, Patient Health Questionnaire 9-item scale; aOR, adjusted Odds Ratio
