# Supplementary material for: Mental Health Changes in US Transgender Adults Beginning Hormone Therapy Via Telehealth: Longitudinal Cohort Study
Source: J Med Internet Res. 2025 Feb 14;27:e64017. doi: 10.2196/64017 (PMC11888058; doi:10.2196/64017)
Supplement: Multimedia Appendix 5 [file jmir_v27i1e64017_app5.pdf]

Multimedia Table 5. Sensitivity Analysis: Association Between Demographic and Mental Health Remission Among Those Without Mental Health Treatment[d], GAS, or Legal Gender Change

	Remission from Depression[a] (n=71)		Remission from Anxiety[b] (n=63)		Resolution from Suicide Ideation[c] (n=42)	
	aOR (95% CI)	*P* value	aOR (95% CI)	*P* value	aOR (95% CI)	*P* value
Age (in years)	1.12 (0.99, 1.25)	0.06	1.01 (0.89, 1.16)	0.84	0.99 (0.86, 1.15)	0.94
Sex Assigned at Birth						
Male	1 [Reference]		1 [Reference]		1 [Reference]	
Female	0.31 (0.10, 1.04)	0.06	0.24 (0.06, 1.02)	0.06	0.48 (0.08, 2.83)	0.42
Non-Latinx White Alone	1.20 (0.38, 3.82)	0.76	0.40 (0.09, 1.87)	0.24	0.47 (0.09, 2.60)	0.39
Insurance Status						
Uninsured	1 [Reference]		1 [Reference]		1 [Reference]	
Insured	0.37 (0.10, 1.42)	0.15	0.81 (0.23, 2.90)	0.75	0.21 (0.04, 1.02)	0.06
Urbanicity of Residence						
City or Suburb	1 [Reference]		1 [Reference]		1 [Reference]	
Rural or Small Town	2.09 (0.65, 6.69)	0.21	1.27 (0.36, 4.48)	0.71	0.89 (0.19, 4.31)	0.89
Education						
Some College or Higher	1 [Reference]		1 [Reference]		1 [Reference]	
High school or less	1.58 (0.56, 4.45)	0.38	0.36 (0.10, 1.32)	0.12	0.94 (0.18, 5.04)	0.94
GAD-7 or PHQ-9 Baseline	0.98 (0.86, 1.13)	0.82	0.93 (0.79, 1.10)	0.40		

[a]All models use Generalized Estimating Equations (GEE) with a binomial family and a logit link function, with clustering at the individual level and controlling for time to follow-up. This model includes those with PHQ-9>=10 at baseline. Remission is defined as a PHQ-9<5 at follow-up.

[b] Model includes those with GAD-7>=8 at baseline. Remission is defined as a GAD-7 score<4 at follow-up.

[c] Model includes those with suicide ideation at baseline. Remission includes those who do not have suicide ideation at follow-up.

[d] Any mental health treatment was defined as using an antidepressant or using talk therapy a year before or during the study period.

Abbreviations: GAS, Gender-Affirming Surgery, GAD-7, Generalized Anxiety Disorder 7-item scale; PHQ-9, Patient Health Questionnaire 9-item scale; aOR, adjusted Odds Ratio
